# Risk factors for postoperative infection of odontogenic cysts associated with mandibular third molar

**DOI:** 10.1186/s40902-020-00248-5

**Published:** 2020-02-18

**Authors:** Jin-woo Kim, Do-hyun On, Jin-yong Cho, Jaeyoung Ryu

**Affiliations:** Department of Oral & Maxillofacial Surgery, Gachon University Gil Medical Center, 21, Namdong-daero 774 beon-gil, Namdong-gu, Incheon, 21565 South Korea

**Keywords:** Odontogenic cysts, Surgical procedures, Surgical wound infection

## Abstract

**Background:**

Odontogenic cysts associated with lower third molar are common. The prognosis for surgical treatment is relatively good. However, postoperative infection discourages the clinicians. Hence, we would like to investigate the factors associated with infection after surgical treatment of cysts associated with the mandibular third molar.

**Methods:**

We retrospectively reviewed the medical and radiographic records of 81 patients who were diagnosed with dentigerous cyst or odontogenic keratocyst and underwent cyst enucleation. The factors affecting postoperative infection were divided into host factor, treatment factor, and cystic lesion factor. To identify the factors associated with postoperative infection, we attempted to find out the variables with significant differences between the groups with and without infection.

**Results:**

A total of 81 patients (64 male and 17 female) were enrolled in this study. There was no statistical relationship about the postoperative infection between all variables (gender, smoking, diabetes mellitus, age, bone grafting, related tooth extraction, previous marsupialization or decompression, type of antibiotics, cortical perforation associated with cystic lesion, preoperative infection, preoperative cyst size).

**Conclusions:**

The results of this study suggest that it is not necessary to avoid bone grafts that are concerned about postoperative infection.

## Background

Intrabony cysts are common lesions of the oral and maxillofacial region [1]. The treatment of cystic lesions associated with mandibular third molar has relatively favorable prognosis [2]. A dentigerous cyst (DC) and odontogenic keratocyst (OKC) are known as common odontogenic cyst in the mandibular third molar region [2–4]. In case which the lesions are similar in size and location, the treatment is similar [5]. Enucleation of cystic lesion with extraction of associated tooth is usually performed, though decompression or marsupialization can also be performed taking into account the patient condition, size and location of the lesion, the potential for pathological fractures, and recurrence [5, 6].

Although surgeons prescribe the antibiotics and educated a patient about the importance of no smoking and maintenance of oral hygiene, surgical site infections may occur irrespective of bone grafts. Unexpected infection symptoms after surgery of cysts may result in poor satisfaction of patients and clinicians. If the statistically significant factors that cause these infections can be identified, treatment predictability can be improved. The purpose of this study was to determine the factors associated with infection after surgical treatment of cysts associated with the mandibular third molar.

## Methods

This study was performed in accordance with the Declaration of Helsinki for medical protocols. The regional Ethical Review Board granted approval (GDIRB2020-041) for the study. We retrospectively reviewed the medical and radiographic records of 81 patients who were diagnosed with DC or OKC and underwent cyst enucleation between 2012 and 2018. Postoperative infection was defined as obvious pain with formation of swelling and pustulation. Patients with postoperative infection signs were classified as infection group and patients who were not as non-infection group. The factors affecting postoperative infection were divided into host factor, treatment factor, and cystic lesion factor. Age, gender, underlying disease such as diabetic mellitus, and smoking of patients were investigated as host factors. Bone graft to the defect after enucleation, type of antibiotics (amoxicillin with clavulanic acid or cephalosporin), marsupialization or decompression before enucleation, the presence or absence of mesial tooth (lower second molar), and its additional treatment such as extraction, endodontic treatment, or apicoectomy were also investigated as treatment factors. Cortical perforation around cyst, size of cystic lesion, preoperative infection, and type of cyst (dentigerous cyst or odontogenic keratocyst) were also evaluated as cystic lesion factors. The size of cystic lesion was calculated using software (INFINITT PACS®, INFINITT Healthcare, Seoul, South Korea) which was measured the longest diameter of the radiolucent lesion.

To identify the factors associated with infection, we attempted to find out the variables with significant differences between the groups with and without infection. Then, we tried to find out which detailed treatment or patient factors influenced the outcome of infection.

## Results

A total of 81 patients (64 male and 17 female) were enrolled in this study (Table 1). The mean age was 47.3 years. Postoperative infection occurred in 21 patients. The average age of the infected patients was 45.3 (22–77) years old and the infection occurred at average 5 weeks (10–93 days) postoperatively. Infection was occurred at 14 patients (20.1%) in DC and 7 patients (50%) in OKC, respectively. There was no statistical significance between the groups with and without the infection by diagnosis (*p = 0.054*). In addition, there was no statistical relationship between all variables (gender, smoking, diabetes mellitus, age, bone grafting, related tooth extraction, previous marsupialization or decompression, type of antibiotics, cortical perforation associated with cystic lesion, preoperative infection, preoperative cyst size). A total of 66 patients did not have tooth extraction related to the cyst (Table 2). No statistical relationship was found between the infection and non-infection groups with regard to the treatment of mesial tooth (extraction, endodontic treatment, and apicoectomy of the lesion-related tooth).

**Table 1:** Description of risk factors affecting infection after the surgery of cyst enucleation

Risk factors	Total (*n* = 81)	Infection group (*n* = 21)	Non-infection group (*n* = 61)	*p* value
Host factors				
Gender (number of males)	64 (79%)	19 (90.5%)	45 (75%)	0.24^†^
Smoking	28 (34.6%)	9 (42.9%)	19 (31.7%)	0.35^†^
Diabetes mellitus	4 (6.7%)	0 (0%)	4 (100 %)	0.34^§^
Age (years)	47.3 ± 14.0	45.3 ± 14.0	48.0 ± 14.1	0.92^‡^
Treatment factors				
Bone grafting	33 (40.7%)	12 (57.1%)	21 (35.0%)	0.12^†^
Related tooth extraction	15 (18.5%)	2 (9.5%)	13 (21.7%)	0.36^†^
Previous decompression	6 (7.4%)	0 (0)	6 (100 %)	0.19^§^
Type of antibiotics (number of cephalosporin)*	21 (25.9%)	8 (38.1%)	14 (23.3%)	0.30^†^
Cystic lesion factors				
Cortex perforation	24 (29.6%)	7 (33.3%)	17 (28.3%)	0.66^†^
Preoperative infection	8 (9.9%)	2 (9.5%)	6 (10.0%)	0.99^§^
Preoperative cyst size (longest diameter of lesion, mm)	20.6 ± 5.21	20.2 ± 4.71	20.7 ± 5.4	0.41^‡^
Type of cyst (number of dentigerous cyst)**	67 (83%)	14 (66.7%)	53 (88.3%)	0.054^†^

^†^Chi-square test

^§^Fisher’s exact test

^‡^Student’s *t* test

*Two kinds of antibiotics were used, amoxicillin with clavulanic acid and cephalosporin

**All subjects were classified as dentigerous cyst or odontogenic keratocyst

**Table 2:** Comparisons between groups according to the treatment method of cystic lesion-related mesial tooth

	Total	Infection group	Non-infection group	*p* value
Extraction of related tooth	81	2	13	0.36^†^
Without extraction of related tooth		19	47	
Within the patients without extraction of related tooth				
Root canal treatment of related tooth	66	11	18	0.24^†^
Without root canal treatment of related tooth		8	29	
Within the patients undergoing root canal treatment of related tooth				
Apicoectomy of related tooth	29	0	2	0.51^§^
Without apicoectomy of related tooth		11	16	

^†^Chi-square test

^§^Fisher’s exact test

## Discussion

In this study, we analyzed the factors of infection after surgical treatment of cystic lesion associated with mandibular third molar. Although infection was more likely to occur after surgical treatment of OKC than DC, the results were not statistically significant (*p* = 0.054). Furthermore, it could be considered to have a no direct effect on postoperative infection because both diseases are developmental cyst not inflammatory origin. Further analysis about infection occurrence for each diagnosis (DC and OKC) was performed with regard to cortical perforation of the cyst lesion, conditions of mesial tooth, and the presence of bone grafting in patient. There was also no statistical significance. The treatment methods or recurrence may vary depending on the diagnosis, but no significant related factors for postoperative infection were found.

In the case of cyst enucleation, bone grafts are preferred to improve bone healing in the bone defects [7]. On the other hand, studies have shown that bone regeneration is possible without bone graft [8, 9]. It was reported to increase bony density up to 97% in relatively large defect (between 20 and 30 mm in size) and 84% in more large defect (between 30 and 50 mm in size) after one year [8]. Therefore, it may be considered that a bone graft should not be done due to a concern about infection of the graft. However, as with other findings, this study suggests that bone graft can be considered when necessary due to the low correlation between bone graft and infection [10].

Most of the studies focusing on the size reduction and recurrence of cystic lesion have been made for decompression procedure before cystectomy. The advantage of decompression procedure of cystic lesion is that the size of the cyst can be reduced to preserve the surrounding anatomical structures and to prevent pathological fractures through minimal facial deformation and bone regeneration during healing period [6, 11, 12]. Although decompression have been shown to improve the prognosis of cyst enucleation in both DC and OKC [13, 14], the results of postoperative infection according to the size of this study were not significant. Although there was no statistical significance, further study is needed to investigate the relation between decompression and postoperative infection, because there was no postoperative infection in 6 patients who underwent decompression procedure before definitive enucleation.

This study found no statistically significant factors associated with postoperative infection of the DC and OKC associated with the mandibular third molar. However, reducing postoperative infection is one of the important things in the surgical treatment. Therefore, it may be necessary to study through an extended population with a well-designed prospective study.

## Conclusions

The results of this study suggest that it is not necessary to avoid bone grafts that are concerned about postoperative infection and there is no justification of decompression to reduce the size of cysts only for the prevention of postoperative infection.

## Data Availability

Not applicable

## Abbreviations

DC: Dentigerous cyst
OKC: Odontogenic keratocyst

## References

[1] Hwang DY, On SW, Song SI (2016). Bone regenerative effect of recombinant human bone morphogenetic protein-2 after cyst enucleation. Maxillofac Plast Reconstr Surg.

[2] Toller P (1967). Origin and growth of cysts of the jaws. Ann R Coll Surg Engl.

[3] Rakprasitkul S (2001). Pathologic changes in the pericoronal tissues of unerupted third molars. Quintessence Int.

[4] Mello FW, Melo G, Kammer PV, Speight PM, Rivero ERC (2019). Prevalence of odontogenic cysts and tumors associated with impacted third molars: a systematic review and meta-analysis. J Craniomaxillofac Surg.

[5] Marker P, Brondum N, Clausen PP, Bastian HL (1996). Treatment of large odontogenic keratocysts by decompression and later cystectomy: a long-term follow-up and a histologic study of 23 cases. Oral Surg Oral Med Oral Pathol Oral Radiol Endod.

[6] Park JH, Kwak EJ, You KS, Jung YS, Jung HD (2019). Volume change pattern of decompression of mandibular odontogenic keratocyst. Maxillofac Plast Reconstr Surg.

[7] Kim HC, Song JM, Kim CJ, Yoon SY, Kim IR, Park BS (2015). Combined effect of bisphosphonate and recombinant human bone morphogenetic protein 2 on bone healing of rat calvarial defects. Maxillofac Plast Reconstr Surg.

[8] Ihan Hren N, Miljavec M (2008). Spontaneous bone healing of the large bone defects in the mandible. Int J Oral Maxillofac Surg.

[9] Ettl T, Gosau M, Sader R, Reichert TE (2012). Jaw cysts - filling or no filling after enucleation? A review. J Craniomaxillofac Surg.

[10] Baek C-H, Park J-H, Kim G-J, Hong J, C-s K, Paeng J-Y (2010). Comparison of healing pattern with or without bone graft after odontogenic cyst enucleation. J Korean Assoc Oral Maxillofac Surg.

[11] Kim T-S, Lee J-H (2010). Spontaneous bone regeneration after enucleation of jaw cysts: a comparative study of panoramic radiography and computed tomography. J Korean Assoc Oral Maxillofac Surg.

[12] Jang C-S, Kim J-W, Yang S-B, Yim J-H, Kim J-Y, Yang B-E (2011). Treatment of large sized cystic lesion of the jaws with specific appliance for decompression: cases report. J Korean Assoc Oral Maxillofac Surg.

[13] Park SH, Kim NK, Kim KH (2008). Postoperative recurrences of odontogenic keratocyst: The behavior and proposal of critical follow-up period. J Korean Assoc Oral Maxillofac Surg.

[14] Park H-S, Song I-S, Seo B-M, Lee J-H, Kim M-J (2014). The effectiveness of decompression for patients with dentigerous cysts, keratocystic odontogenic tumors, and unicystic ameloblastoma. J Korean Assoc Oral Maxillofac Surg.

